# Integrative analysis of m3C associated genes reveals METTL2A as a potential oncogene in breast Cancer

**DOI:** 10.1186/s12967-022-03683-2

**Published:** 2022-10-20

**Authors:** Shuai Wang, Huiting Li, Jiheng Liu, Qianqian Zhang, Wei Xu, Juanjuan Xiang, Li Fang, Ping Xu, Zheng Li

**Affiliations:** 1 NHC Key Laboratory of Carcinogenesis and Hunan Key Laboratory of Cancer Metabolism, Hunan Cancer Hospital and the Affiliated Cancer Hospital of Xiangya School of Medicine, Central South University, Changsha, Hunan China; 2 Department of Hematology and Oncology, First Hospital of Changsha, Changsha, Hunan China; 3 Departments of Respiratory and Critical Care Medicine, Peking University Shenzhen Hospital, Shenzhen, Guangdong China

**Keywords:** N3-methylcytidine, METTL2A, METTL2B, METTL6, METTL8, Breast cancer

## Abstract

RNA methylation modifications, especially m6A mRNA modification, are known to be extensively involved in tumor development. However, the relationship between N3-methylcytidine (m3C) related genes and tumorigenesis has rarely been studied. In this research, we found that m3C-related genes were expressed at different levels and affected patients’ prognosis across multiple cancer types from The Cancer Genome Atlas and multi-omics levels. Importantly, methyltransferase-like proteins 2A (METTL2A) had a high amplification frequency (~ 7%) in patients with breast invasive carcinoma (BRCA), and its overexpression was an independent predictor of poor overall survival. Enrichment analysis of associated genes revealed that METTL2A may activate DNA synthesis and cell proliferation pathways in BRCA cells. Through drug sensitivity analysis, Trifluridine, PD407824, and Taselisib were shown to be effective drugs for METTL2A-positive BRCA patients. Overall, our research conducts a holistic view of the expression level and prognostic signature of m3C-related genes with multiple malignancies. Importantly, METTL2A has been intensely explored as a potential oncogene in BRCA, to aid the development of potential drug agents for precision therapy in breast cancer patients.

**Supplementary Information:**

The online version contains supplementary material available at 10.1186/s12967-022-03683-2.

## Background

With decades of epitranscriptomal studies, it is increasingly clear that the covalent modifications in RNAs are a crucial additional aspect of gene regulation. It is convinced that 163 post-transcriptional modifications of RNA contribute strongly to the diversity of functions fulfilled by RNA molecules, especially during the process of tumor progression [1]. Methylations are among the most common RNA modifications, which include N6-methyladenosine (m6A) [2], N1-methyladenosine (m1A) [3], N5-methylcytidine (m5C) [4], and N3-methylcytidine (m3C) [5]. Various methylation modifications have a wide range of effects on the RNA secondary structure folding, stability, and function, which influence the physiological processes and are closely related to numerous human diseases [1].

The m6A modification, one of the most well-known mRNA modifications, is extensively involved in all sorts of tumor development [2]. In contrast, the relationship between m3C modification and tumorigenesis has very rarely been studied. The 3-methylcytidine modification at position 32 (m3C32) is discovered in fission yeast with two enzymes, Trm140 and Trm141, catalyze tRNA^Thr^ and tRNA^Ser^ m3C32 modification, respectively [6]. The N6-threonylcarbamoyladenosine (t6A) or N6-isopentenyladenosine (i6A) modification at position 37 (t6A37 or i6A37) of yeast tRNA^Thr^ or tRNA^Ser^ function as a key determinant for the m3C process [7]. While the m3C modification for tRNA^thr^/tRNA^Arg^ and tRNA^ser^ are catalyzed by Mettl2 and Mettl6 in mice, respectively. In addition, the deletion of Mettl8 has little influence on the abundance of tRNAs which supports the hypothesis that Mettl8 is an mRNA m3C methyltransferase rather than a tRNA [8]. In human cells, N3-methylcytidine of RNA can be catalyzed by methyltransferase-like proteins (METTLs) that contain 4 members of METTL2A, METTL2B, METTL6, and METTL8 [8]. METTL2A and METTL2B are two homologs of Mettl2 with only six amino acids differ. With G35 and t6A at position 37 (t6A37) on the anticodon loop, METTL2A catalyzes the m3C32 in human tRNA^Thr^, while there is little m3C32 modification activity presented by METTL2B. METTL6 interacts with seryl-tRNA synthetase (SerRS, encoded by SARS1) to mediate the biogenesis of m3C32 modification in human tRNA^Ser^ [9]. In contrast to the preceding three cytoplasmic m3C32-modifying enzymes, it had been established that METTL8 functioned in mitochondria [10, 11].

There were several studies figuring out the vital important relationship between METTL6 and the prognostic of malignant tumor patients. It was convinced that knockdown of METTL6 could significantly decrease sensitivity of lung cancer cells to cisplatin [12]. A bioinformatics analysis revealed that METTL6 tended to amplify in luminal breast tumors with highly proliferative capacity [13, 14]. Recently, it is demonstrated that the deletion of METTL6 showed a significant inhibition on the proliferation of liver cancer cell lines and the high expression of METTL6 in malignant patients tended to indicate a poor prognostic [15, 16]. The m3C32 modification of mitochondria tRNA^Ser^ and tRNA^Thr^ were catalyzed by the mitochondrial protein METTL8 and the high expression of METTL8 contributed an enhanced respiratory chain activity in pancreatic cancer [10]. The alternative mRNA splicing research reported that one of the isoforms of METTL8, METLL8-iso1, had been investigated to be transported to mitochondrial with the assistance of its N-terminal mitochondrial targeting sequence [11]. Aside from the classical m3C32 modification of mitochondrial tRNAs, upregulated of METTL8 by Yin Yang 1 (YY1) transcription factor mediated the m3C modification on the mRNA of AT-rich interactive domain-containing protein 1A and attenuated its translation, which induced the migration of breast cancer cell lines [17]. In addition, the SUMOylated METTL8 induces the R-loop formation and promotes tumorigenesis in colorectal cancer [18, 19]. Nevertheless, the functions and mechanisms of m3C enzymes in cancer progression remain poorly understood.

Herein, we conducted the landscape analysis of four m3C enzymes' expressions and their relationship with prognosis across multiple cancer types. We also analyzed the DNA copy numbers and mutations of the four m3C associated genes using multi-omic data from The Cancer Genome Atlas (TCGA). Furthermore, we determined the correlation of METTL2A expression with clinical parameters of patients with breast cancer and analyzed related signaling pathways and potential therapeutic agents of METTL2A in BRCA patients. Finally, we detected METTL2A expression in tumor tissues of BRCA using immunohistochemistry. The data and analyses suggest that m3C associated genes, especially METTL2A, play a key function and are tightly linked to the development of various cancers.

## Method

### Analysis of m3C associated genes in pan-cancer

The gene expression data and survival profiles of 33 cancers from the University of North Carolina TCGA genome characterization center was downloaded from the UCSC Xena (https://xenabrowser.net/) in log2(x + 1) transformed FPKM normalized count form [20, 21]. Because some cancer types are lack corresponding normal tissue in TCGA, we carried out the differential expression analysis between tumor tissue and normal tissue in 24 cancer types. Gene id was transformed to gene symbol using the annotation file downloaded from the NCBI website (https://www.ncbi.nlm.nih.gov/) [22]. The genes with a *P* < 0.05 were assumed to be significantly variational genes in tumor tissue or among various subtypes.

The survival analysis of the four m3C associated genes in all TCGA tumor patients was carried out using the GEPIA2 database (http://gepia2.cancer-pku.cn) [23]. Then the cox proportional hazard ratio and Kaplan–Meier curve were obtained from this website. The cBioPortal for Cancer Genomics (https://www.cbioportal.org/) provided a convenient tool to analyze multidimensional cancer genomics data [24, 25]. A total of 10967 TCGA patients across 32 studies (lack of READ) were included in the mutation and copy number variation analysis with the keyword "TCGA pan-cancer atlas". The OncoPrint module of the cBioPortal database displayed a concise and compact graphical summary of genomic alterations in the four m3C associated genes across 32 cancer types. The package perturbation clustering for data integration and disease subtyping (PINSPlus) was utilized to analysis the classification of cancer into various subtypes [26, 27]. The number of clusters was set to be 3 to keep the consistency across various tumors. The clustering algorithm was k-means and the perturbation method was noise for default. The subtypes with different survival profiles were presented on the K-M plot and Pheatmap plot.

### Gene expression profiling analysis of m3C associated genes in BRCA

Then the breast invasive carcinoma cohort was enrolled to explore the genomic alterations and their relation to the expression of the four m3C associated genes. The expression data and clinical data of 719 BRCA positive patient cohort were also downloaded from the UCSC Xena for predictive analysis [20, 21]. The gene expression data and clinical information of the GSE3744 (including 7 normal samples and 40 BRCA tumor samples based on GPL570) [28, 29], GSE1456 (including 159 BRCA patients based on GPL96 and GPL97) [30, 31], GSE3494 (including 251 BRCA patients based on GPL96 and GPL97), GSE4922 (including 289 BRCA patients based on GPL96 and GPL97) [32], and GSE6532 (including 327 BRCA patients based on GPL96, GPL97 and 87 BRCA patients based on GPL570) [33], datasets were downloaded from the Gene Expression Omnibus (GEO) (https://www.ncbi.nlm.nih.gov/geo/) [34]. The expression data were all normalized using the robust multi-array average method. Using the annotation file of GPL96, GPL97, and GPL570, gene ids were transformed to gene symbols. Finally, five validation BRCA cohorts were established. Samples with missing clinical features were excluded when analyzing. In addition, the UALCAN tool was utilized to conduct METTL2A protein expression analysis with data from Clinical Proteomic Tumor Analysis Consortium (CPTAC) dataset [35, 36].

### Immunohistochemistry and scoring

Samples from four patients with histological diagnosis of BRCA at the First Hospital of Changsha, China, during 2020, were retrospectively collected. The TNM stage were categorized according to the NCCN Guidelines Version 1.2022 Breast Cancer. The use of patient information and tissues was sanctioned by the Ethics Committee of the First Hospital of Changsha. The detail information for the breast cancer tissues was included in the raw data for Fig. 6.

Immunohistochemistry (IHC) staining was performed with an immunohistochemical kit DAB chromogenic agent (Servicebio, G1211). Briefly, formalin-fixed, paraffin-embedded tumors were sectioned, and slides were deparaffinized using xylenes and rehydrated in a graded alcohol system. Then the tissue slides were sequentially treated with citric acid (PH6.0) antigen retrieval buffer for antigen retrieval and 3% H2O2/MeOH for blocking endogenous peroxidase activity. After being blocked with 3% bovine serum albumin (BSA), the sections were incubated with anti-METTL2A antibody and treated with secondary antibody (HRP labeled) from the corresponding species of primary antibody. This step was followed by DAB chromogenic reaction and nucleus counterstaining with hematoxylin stain solution. Finally, the stained sections were dehydrated, sealed and visualized. The staining intensities of protein were analyzed with ImageJ (https://imagej.net/Fiji).

### Gene ontology enrichment analysis (GO) and gene set enrichment analysis (GSEA)

Based on the METTL2A correlated genes, GO enrichment analysis was conducted through the package “clusterProfiler” in R.4.0.0 [37]. The top 30 of enriched pathways with *adj P* < 0.05 were assumed to be enriched GO pathways and presented on the histogram. Additionally, GSEA analysis was carried out in the TCGA BRCA cohort to investigate the tumor hallmarks that were more common in the METTL2A high expression subgroup compared to the METTL2A low expression subgroup (divided according to the median expression of METTL2A). The annotated gene set was selected (hallmark gene sets) as the reference gene set. Gene set permutations were set to 1000 times to identify significantly different pathways. The GSEA pathways with nominal *P* < 0.05 and FDR < 0.05 were identified as a significantly enriched hallmark pathway of GSEA.

### Drug sensitivity analysis

The drug sensitivity data of cell lines were acquired from the PRISM Repurposing dataset (https://www.theprismlab.org/) and the Genomics of Drug Sensitivity in Cancer (GDSC) dataset (https://www.cancerrxgene.org/). In addition, the expression data of the PRISM cell lines was downloaded from the DepMap portal (https://depmap.org/portal/) while the GDSC cell lines from the GDSC dataset. Both projects provided the area under the dose–response curve (area under the curve-AUC) values as a measure of drug sensitivity, and a lower AUC value indicates increased sensitivity to treatment. All compounds with more than 20% missing data were excluded from this study. Then K-nearest neighbor (k-NN) imputation was applied to impute the missing AUC values. Finally, sensitivity data for 981 compounds over 22 BRCA cell lines from PRISM and 262 compounds over 46 BRCA cell lines from GDSC were included in this research.

### Statistical analysis

All statistical analyses were performed in GraphPad Prism software and R statistical software (v4.0.0, R Core Team, R Foundation for Statistical Computing, Vienna, Austria). Before the statistical test, the normality test and the homogeneity test of variance were performed. Comparison of a continuous variable in two or multiple groups was performed using a parametric test (Student’s t-test or analysis of variance) if the variable was normally distributed or a nonparametric test (Wilcoxon rank-sum test or Kruskal–Wallis test). Correlation between two continuous variables was measured by either Pearson’s r correlation or Spearman’s rank-order correlation. The hazard ratio (HR) was estimated using a Cox regression model. When survival analysis was carried out using Kaplan–Meier methods, the patients with OS missing or OS time < 30 days had been deleted to reduce statistical bias, and the log-rank test was used to determine the statistical significance of differences. For all statistical analyses, a two-tailed *P* < 0.05 was considered statistically significant. All the analyses process had been uploaded to the github website in a R script version (https://github.com/Hannisal/M3C.git).

## Results

### Widespread changes of m3C associated genes expression in multiple cancer types

**Fig. 1:**
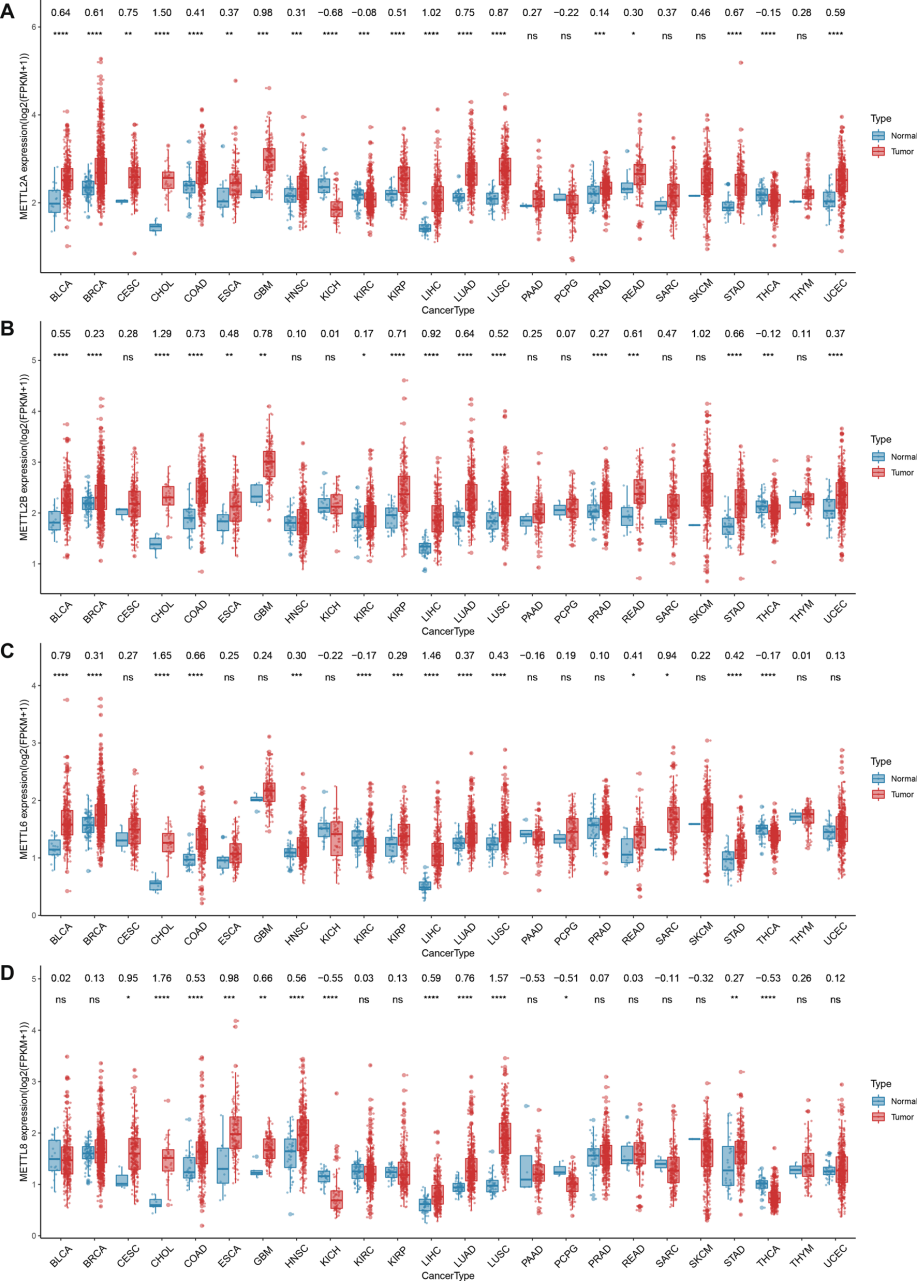
Aberrant expression of m3C associated genes in cancer. Expression alterations of m3C related genes between tumor (red) and normal(blue) tissue samples across multiple cancer types** a** METTL2A, **b** METTL2B, **c** METTL6, and **d** METTL8. At the top of each graph is the log Fold Change value and *P* value. *P < 0.05, **P < 0.01, ***P < 0.001, ****P < 0.0001

The differential expression analysis of METTL2A, METTL2B METTL6 and METTL8 was conducted between tumor tissue and normal tissue in 24 different cancer types. The m3C associated genes showed aberrant expression in 17 cancer types for METTL2A (Fig. 1a), 15 cancer types for METTL2B (Fig. 1b), 14 cancer types for METTL6 (Fig. 1c) and 13 cancer types for METTL8 (Fig. 1d). As shown in Fig. 1, the expression levels of all m3C associated genes were higher than the corresponding normal tissues in the tumor tissue of Cervical squamous cell carcinoma and endocervical adenocarcinoma, Cholangiocarcinoma, Colon adenocarcinoma, Esophageal carcinoma, Head and Neck squamous cell carcinoma, Liver hepatocellular carcinoma (LIHC), Lung adenocarcinoma (LUAD), Lung squamous cell carcinoma and Stomach adenocarcinoma (STAD). In contrast, there was down-regulation of these four genes in Kidney Chromophobe (KICH), Kidney renal clear cell carcinoma (KIRC), and Thyroid carcinoma Compared to METTL8, the other three genes (METTL2A, METTL2B and METTL6) exhibited a more homogenized co-expression in various cancer types.

### The prognostic role of m3C associated genes in multiple types of cancer

**Fig. 2:**
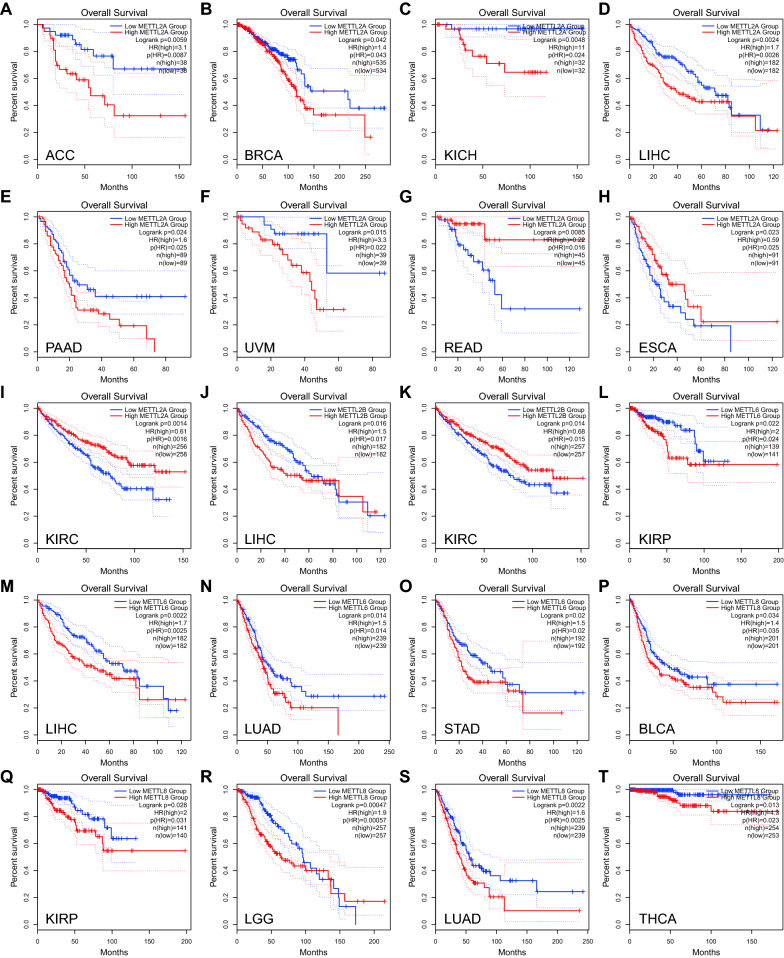
Prognostic value of m3C associated genes in cancer. **a**–**j** Survival curves for METTL2A. **k** Survival curve for METTL2B. **l**–**o** Survival curves for METTL6. **p**–**t** Survival curves for METTL8. **K**–**M** plots depict the survival curves for each m3C related gene in multiple cancer types and patients were divided into the high expression group (red) and low expression group (blue) according to the gene expression. The ones with Logrank’s *P* < 0.05 are presented in this figure

**Fig. 3:**
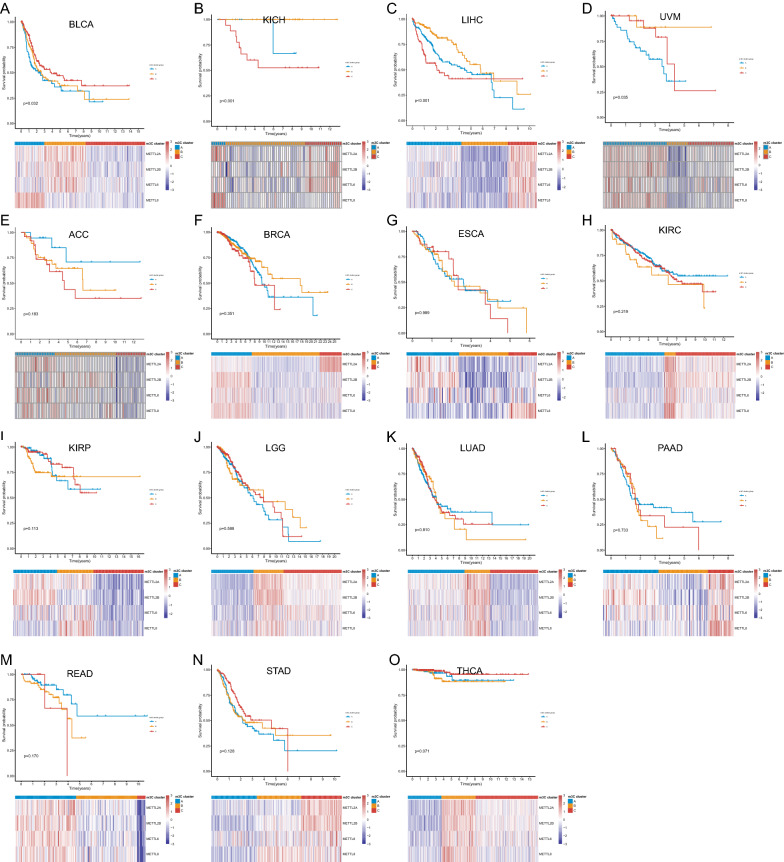
Perturbation cluster for disease subtyping analysis (PINSPlus) of m3C associated genes in cancer. The survival curves for subtypes of cancers and the pheatmap plot for m3C associated genes were presented respectively

To study the prognostic role of m3C associated genes in multiple types of cancer, the Kaplan–Meier methods and Cox regression were utilized in the TCGA cohort. The Cox proportional hazard ratio and Kaplan–Meier curve assay results were consistent in terms of the prognostic trend in multiple cancers (Fig. 2, Table 1). In LIHC, METTL2A, METTL2B and METTL6 high expression was associated with poor prognosis. Contrarily, METTL2A and METTL2B low expression were related to poor prognosis in KIRC. Respectively, METTL2A high expression was related to poor prognosis in Adrenocortical carcinoma, Breast invasive carcinoma (BRCA), KICH, Pancreatic adenocarcinoma (PAAD) and Uveal Melanoma (UVM), while low expression was related to poor prognosis in Esophageal carcinoma and Rectum adenocarcinoma (READ). METTL6 high expression was associated with poor prognosis in LIHC, LUAD and STAD. METTL8 high expression was related to poor prognosis in Bladder Urothelial Carcinoma (BLCA), Kidney renal papillary cell carcinoma, Brain Lower Grade Glioma, LUAD and Thyroid carcinoma. Among the 15 cancer types, there was at least one m3C associated gene that was significant related to the prognosis. We carried out the PINSPlus cluster analysis to find out different subtypes of cancer and presented their survival profiles. The results showed that abnormal expression of m3C associated genes may be used as candidate markers for molecular subtypes of BLCA, KICH, LIHC and UVM (Fig. 3).

**Table 1:** The cox proportional hazard ratio and corresponding *P* value for m3C associated genes across multiple cancer types

Overall Survival	METTL2A		METTL2B		METTL6		METTL8	
Cancer Type	HR (high)	P (HR)	HR (high)	P (HR)	HR (high)	P (HR)	HR (high)	P (HR)
ACC	**3.1**	**0.0087**	1.9	0.12	2	0.085	1.8	0.15
BLCA	1.2	0.24	1.3	0.072	1.1	0.58	**1.4**	**0.035**
BRCA	**1.4**	**0.043**	0.95	0.77	1.1	0.43	1.2	0.33
CESC	1.3	0.22	1.5	0.07	1	0.97	1.4	0.18
CHOL	0.85	0.73	0.99	0.98	0.44	0.11	0.47	0.13
COAD	0.84	0.49	0.61	0.056	1.5	0.09	0.82	0.43
DLBC	1.5	0.55	0.72	0.66	0.64	0.54	1.9	0.38
ESCA	**0.59**	**0.025**	0.83	0.44	1.2	0.49	0.8	0.35
GBM	0.9	0.58	0.92	0.66	0.77	0.15	0.98	0.93
HNSC	1.1	0.34	1.1	0.41	0.9	0.46	1.2	0.18
KICH	**11**	**0.024**	8.00E + 08	1	6.70E + 08	1	2.3	0.24
KIRC	**0.61**	**0.0016**	**0.68**	**0.015**	0.88	0.39	0.75	0.064
KIRP	1.1	0.84	1	0.97	**2**	**0.024**	**2**	**0.031**
LAML	1.3	0.3	0.64	0.12	1.2	0.49	1.2	0.53
LGG	1.2	0.3	1.4	0.052	0.86	0.4	**1.9**	**0.00057**
LIHC	**1.7**	**0.0026**	**1.5**	**0.017**	**1.7**	**0.0025**	1.3	0.1
LUAD	1.3	0.097	1.1	0.48	**1.5**	**0.014**	**1.6**	**0.0025**
LUSC	0.87	0.31	1	0.79	1	0.93	0.9	0.45
MESO	1.2	0.38	1.1	0.68	1.1	0.58	1.6	0.053
OV	1	0.76	1.2	0.18	1.1	0.6	0.84	0.17
PAAD	**1.6**	**0.025**	1.2	0.42	1.1	0.76	1.4	0.14
PCPG	2	0.42	2.7	0.26	1.6	0.6	1.4	0.69
PRAD	2.7	0.15	2.9	0.13	1.2	0.73	1.7	0.41
READ	**0.22**	**0.016**	0.44	0.12	0.37	0.06	0.65	0.37
SARC	0.71	0.094	0.91	0.63	1.3	0.19	0.79	0.26
SKCM	1.1	0.53	1.1	0.54	0.99	0.93	1.1	0.54
STAD	0.82	0.22	1	0.77	**1.5**	**0.02**	1	0.79
TGCT	2.2	0.5	0.95	0.9	1	0.98	0.34	0.35
THCA	1.6	0.39	2.3	0.13	1.6	0.34	**4.3**	**0.023**
THYM	1.5	0.61	1.4	0.65	0.72	0.66	2.8	0.21
UCEC	1.1	0.78	0.89	0.74	0.87	0.7	1.2	0.65
UCS	0.56	0.098	0.84	0.6	1	0.96	1.1	0.73
UVM	3.3	**0.022**	1.7	0.24	0.57	0.21	2	0.14

Significant P values are highlighted in bold

### The variation on genetic alteration status of m3C associated genes in multiple types of cancer

**Fig. 4:**
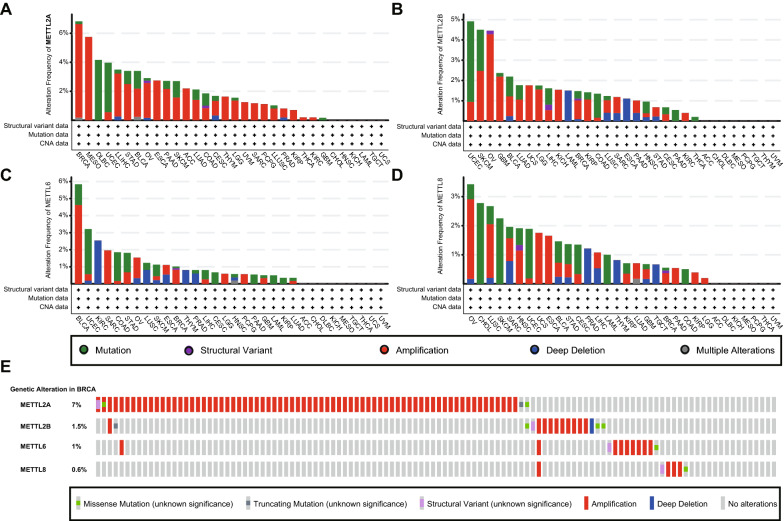
Genetic alteration status of m3C associated genes in cancer. **a**–**d** The genetic alteration status (mutation, structural variant, amplification, deep deletion, and multiple alterations) for **a** METTL2A, **b** METTL2A, **c** METTL6, and **d** METTL8 in multiple cancer types. **e** The presentation of m3C related genes’ alteration status in BRCA, containing missense mutation, truncating mutation, structural variant, amplification, and deep deletion

We observed the genetic alteration status of m3C associated genes in different cancer types of the TCGA cohorts. As shown in Fig. 4, The top three cancer types with genetic alteration of METTL2A is BRCA(7%), Mesothelioma (6%), and Lymphoid Neoplasm Diffuse Large B-cell Lymphoma (4%)(Fig. 4a). The top three cancer types with genetic alteration of METTL2B is Uterine Corpus Endometrial Carcinoma (5%), Skin Cutaneous Melanoma (2%), and Ovarian serous cystadenocarcinoma (4%) (Fig. 4b). The frequency of METTL6 alteration is the highest in BLCA (4.5%), Uterine Corpus Endometrial Carcinoma (3%), and KIRC (2.5%) (Fig. 4c). METTL8 show lower alteration frequency of 3% in Ovarian serous cystadenocarcinoma, 3% in Cholangiocarcinoma, and 2% in Lung squamous cell carcinoma (Fig. 4d). Interestingly, the main genetic alteration type of METTL2A is amplification in BRCA (Fig. 4e). Together, these results reveal the conserved heterogeneity of m3C associated genes in multiple cancers, showing that m3C associated gene disorders could play an important role in different cancers.

### METTL2A protein expression was upregulated in BRCA tissues

**Fig. 5:**
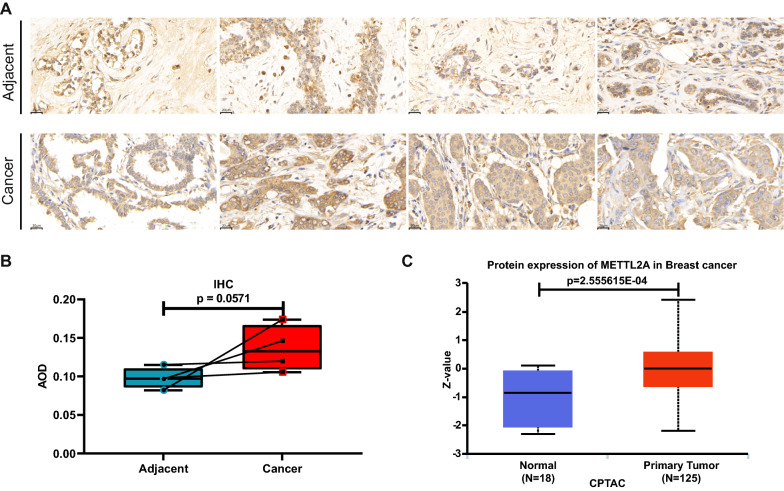
METTL2A protein expression in BRCA tissues.** a** Immunohistochemical staining of BRCA tissues and paired adjacent normal tissue. **b** Mann–Whitney test for the different average optical density value between tumor and adjacent normal tissue. **c** The different protein expression of METTL2A in breast cancer from Clinical Proteomic Tumor Analysis Consortium (CTPAC) database

The significant expression change, the role in prognosis and amplification on genetic alteration status of METTL2A in BRCA made it attractive. Next to determine the protein expression level of METTL2A in BRCA tissues, we used IHC staining and CPTAC database. The IHC result showed that METTL2A protein expression was higher in tumor tissues compared to adjacent noncancerous tissues (Fig. 5a, b). Consistently, the protein level of METTL2A was significantly higher in BRCA tumor tissues compared with normal tissues in CPTAC database (Fig. 5c). Altogether, these results suggest that METTL2A might be an oncogene and could play an important role in the progression of BRCA.

### Univariate and multivariate analysis of METTL2A in BRCA

The univariate and multivariate analysis were ultilized to investigated the independent prognostic value of METTL2A in the BRCA cohort (Table 2). Based the 719 BRCA patients with expression data and corresponding clinical profiles from TCGA cohort, the patients with clinical features as male, TX, NX, MX, Indeterminate, Equivocal and OS.time < 30 were removed for the cox proportional hazard regression analyses. Finally, in 681 BRCA patients, the Cox proportional hazard regression model was used to evaluate the impact of METTL2A expression and other clinical feature factors on survival. Univariate analysis showed that age (HR, 2.03; 95% CI 1.34–3.07; *P* = 8.00E-04), T stage (HR, 1.43; 95% CI 1.11–1.84; *P* = 0.0052), N stage (HR, 1.52; 95% CI 1.22–1.9; *P* = 2.00E-04), M stage (HR, 5.79; 95% CI 2.99–11.21; *P* < 0.001), and METTL2A expression (HR, 1.4; 95% CI 1–1.97; *P* = 0.0496) were important predictors of survival. In addition, multivariate analysis results showed that the high expression of METTL2A was an important independent predictor of poor overall survival (HR, 1.44; 95% CI 1–2.06; *P* = 0.0482).

**Table 2:** The Univariate and Multivariate Analysis for METTL2A in BRCA

	Univariate analysis			Multivariate analysis		
	Hazard ratio	CI 95	P Value	Hazard ratio	CI 95	P value
BRCA						
Age	2.03	1.34–3.07	**8.00E−04**	2.02	1.31–3.11	**0.0014**
T stage	1.43	1.11–1.84	**0.0052**	1.18	0.89–1.56	0.2485
N stage	1.52	1.22–1.9	**2.00E−04**	1.28	0.98–1.68	0.0746
M stage	5.79	2.99–11.21	** < 0.0001**	2.53	1.11–5.74	**0.0269**
ER status	0.95	0.59–1.53	0.8301			
PR status	0.83	0.55–1.27	0.3992			
HER2 status	0.99	0.54–1.81	0.9649			
METTL2A	1.4	1–1.97	0.0496	1.44	1–2.06	**0.0482**

Significant P values are highlighted in bold

### The association between METTL2A expression and clinical parameters of BRCA

To further identify the role of METTL2A in BRCA, we further explored METTL2A expression and clinical features of BRCA using GEO data (Additional file 1: Fig. S1). From the GSE3744 data analysis, we observed METTL2A was significantly overexpressed in breast cancer tissues. In GEO BRCA cohorts including GSE1456 (n = 159), GSE4922 (n = 242) and GSE6532 (n = 238), the K-M plot revealed that BRCA patients with METTL2A high expression exhibited poor prognosis, which was consistent with the TCGA BRCA cohort.

**Fig. 6:**
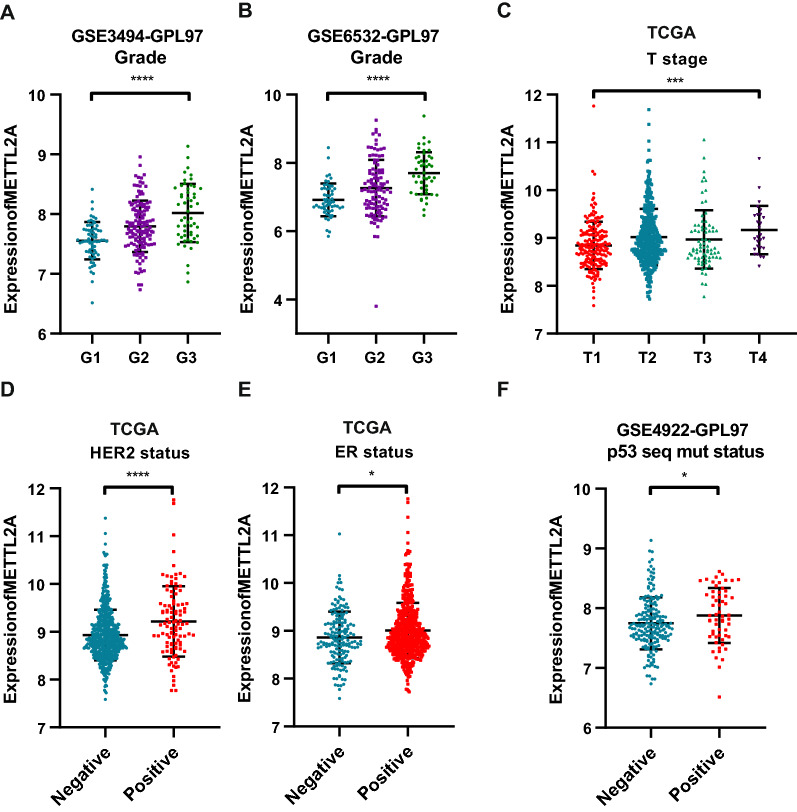
Relationship between METTL2A expression and clinicopathological parameters in BRCA. Distribution of METTL2A expression stratified by **a, b** Grade, **c** T stage,** d** HER2 status, **e** ER status, and** f** P53 mutation status. *P < 0.05, **P < 0.01, ***P < 0.001, ****P < 0.0001

Considering the bias coming with gender, only female BRCA patients in TCGA cohort (n = 711) were included for further analysis. Table 3 summarizes the correlation between METTL2A expression level and various clinical features in BRCA patients. METTL2A high expression was more prevalent in patients with advanced T stage and high grade (Fig. 6a–c). There was no significant relationship between METTL2A expression and age, N and M stage (Additional file 2: Fig. S2a–d). The expression of METTL2A was higher in HER2 positive and ER positive breast cancer patients (Fig. 6d, e). But there was no significant difference in the PR subgroup **(**Additional file 2: Fig. S2e–g). In addition, p53 mutation-positive patients showed higher METTL2A expression (Fig. 6f). Taken together, our results reveal that higher METTL2A expression was associated with higher T stage, higher grade, P53 mutation positive, HER2 positive or ER positive status in BRCA patients.

**Table 3:** The Baseline table for the BRCA cohort from TCGA

Characteristics	Total (%)	METTL2A expression		χ^2^	P	Method
		High	Low			
Age				0.1134998	0.73619431	Chi-square
< = 58	362(50.91)	178	184			
> 58	349(49.09)	177	172			
T stage				16.70803579	**0.000811486**	Chi-square
T1	186(26.16)	73	113			
T2	422(59.35)	231	191			
T3	76(10.69)	33	43			
T4	27(3.8)	18	9			
N stage				1.932257547	0.586585254	Chi-square
N0	350(49.23)	168	182			
N1	237(33.33)	121	116			
N2	85(11.95)	43	42			
N3	39(5.49)	23	16			
M stage				2.13343323	0.144117645	Chi-square
M0	699(98.31)	346	353			
M1	12(1.69)	9	3			
ER status				1.292338005	0.255617533	Chi-square
Negative	164(23.07)	75	89			
Positive	547(76.93)	280	267			
PR status				0.182133618	0.669546019	Chi-square
Negative	230(32.35)	118	112			
Positive	481(67.65)	237	244			
HER2 status				17.25954201	**3.26E-05**	Chi-square
Negative	607(85.37)	283	324			
Positive	104(14.63)	72	32			

Significant P values are highlighted in bold

### Identification of METTL2A-related signaling pathways and potential therapeutic agents in BRCA

**Fig. 7:**
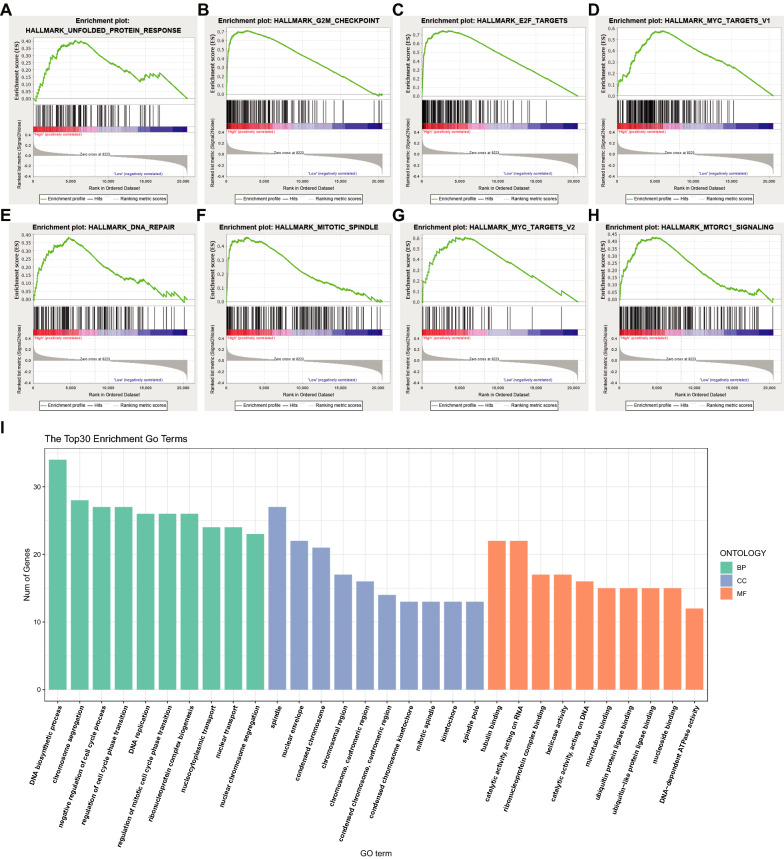
METTL2A related genes enriched signaling pathways. **a**–**h** Gene set enrichment analysis (GSEA) enrichment map of METTL2A related genes. **i** Enrichment analysis of METTL2A related genes by Gene Ontology

Based on the expression of METTL2A, GSEA analysis was performed in the TCGA cohort. The results revealed that eight tumor hallmarks were enriched in the METTL2A high-expression subgroup, which included UNFOLDED_PROTEIN_RESPONSE, MYC_TARGETS_V1, G2M_CHECKPOINT, E2F_TARGETS, DNA_REPAIR, MYC_TARGETS_V2, MTORC1_SIGNALING AND MITOTIC_SPINDLE (Fig. 7a–h). Meanwhile, the genes with r > 0.3 and *P* < 0.05 in TCGA were included for GO enrichment analysis to study METTL2A related GO pathway in breast tumor tissue. The results showed that several cell cycle-related pathways were enriched, such as chromosome segregation, DNA biosynthetic process, spindle, chromosomal region, catalytic activity, acting on RNA, tubulin binding to name a few (Fig. 7i). The consistency of our results indicates that breast cancer cells with high METTL2A expression could be active in proliferation and DNA damage response pathways.

**Fig. 8:**
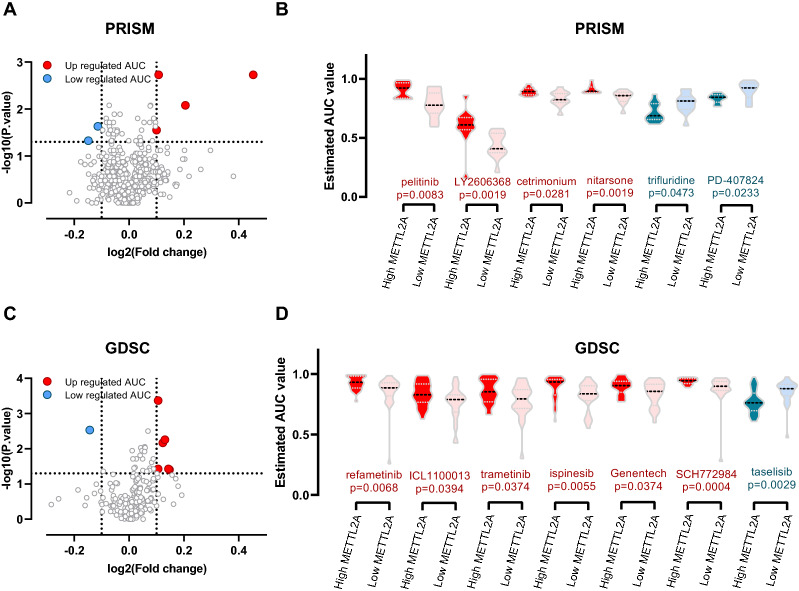
The potential therapeutic agents for METTL2A high expression BRCA patients. Volcano plot and violin plot of differential drug response analysis in **a, b** PRISM and **c, d** GDSC between METTL2A high expression (top decile) and low expression (bottom decile) group

The drug sensitivity analysis was performed using GDSC and PRISM-derived drug response data. First, differential drug response analysis between METTL2A high expression (top decile) and low expression (bottom decile) groups was conducted to identify compounds with lower estimated AUC values in the METTL2A high expression group (log2FC < − 0.10 and *P* < 0.05) (Fig. 8a–d). The therapeutic effect of 13 agents was identified in GDSC and PRISM, respectively. It was shown that Trifluridine, PD407824 and Taselisib were potential therapeutic agents for METTL2A high expression BRCA patients. In contrast, patients with METTL2A high expression demonstrated resistance to pelitinib, LY2606368, cetrimonium, nitarsone, refametinib, ICL1100013, trametinib, ispinesib, Genentech and SCH772984. Trifluridine replaces thymine in DNA replication and directly mixes into the DNA double strands [38]. PD407824 is a CHK1 and WEE1 inhibitor [39] and Taselisib is a potent PI3K inhibitor [40]. Together with the results from METTL2A associated gene network, we speculated these drugs may be as effective treatment of BRCA patients with the over-expression of METTL2A.

## Discussion

M3C modification was first found in Saccharomyces cerevisiae [5]. In S. cerevisiae, Trm140 and Trm141 catalyze m3C formation in tRNA^Thr^ and tRNA^Ser^, respectively [7]. Four Trm140 and Trm141 homologs have been discovered in humans: METTL2A, METTL2B, METTL6, and METTL8. METTL2A and METTL2B were shown to modify m3C at position 32 of tRNA^Thr^ isoacceptors and tRNA^Arg^(CCU) [8]. Here, we found significant correlation between m3C associated genes and tumor malignancy in transcription and genetic alteration in a variety of tumor types. Consistent with the former researches [12, 15], the negative relationship between the expression of METTL6 and the prognostic of LUAD and LIHC had been verified in the TCGA cohort. In addition, the four m3C associated genes shared the same expression pattern among several cancer types (containing BRCA, LIHC, LUAD, PAAD, and READ), which implied that they all function as oncogenes in these cancers. As described above, METTL8 had been proved to be an oncogene in BRCA with its direct m3C modification on the mRNA of ARID1A [17], while METTL6 shown a tendency of amplification in malignant breast cancer patients [13]. Interestingly, compared with METTL8 and METTL6, differential expression of METTL2A in BRCA tumor tissues and adjacent normal tissues was more significantly.

Expectedly, METTL2A high expression correlated with poorer survival outcomes of BRCA patients in the TCGA and multiple GEO cohorts. Based on the genetic alteration analysis of m3C associated genes in BRCA, it was convinced that there was a higher genetic alteration of METTL2A with the rate of amplification up to 7% which was about seven times higher than METTL6 [13]. Therefore, it was further proved that METTL2A played a more important oncogene role in BRCA than the other m3C associated genes. Furthermore, we uncovered that the expression level of METTL2A was significantly positively related to T stage, grade, P53 mutation status, HER2, and ER positive status. Using IHC and CPTAC, we found that the protein level of METTL2A was up-regulated in BRCA tumor tissues. These results suggest that METTL2A might be an oncogene that plays an important role in BRCA progression. However, larger sample sizes and further research would be necessary for validation and expanding on our research.

The related Hallmark signaling pathway of METTL2A in BRCA was analyzed by the GSEA and GO database. The results showed that G2M_CHECKPOINT, E2F_TARGETS, DNA_REPAIR, and proliferation and DNA damage response pathways were correlated with the high expression of METTL2A. These results suggested that METTL2A may affect cell cycle and proliferation of cells to promote the progression of BRCA. In addition, Trifluridine, PD407824 and Taselisib were considered to be potential therapeutic agents for METTL2A high expression BRCA patients. Taselisib is currently in Phase III trials for postmenopausal women with estrogen receptor-positive (ER +) breast cancer [41] and non-small cell lung cancer (NSCLC) [42]. Taselisib is a potent PI3K inhibitor that inhibits the activity of PIK3CA, PIK3CB and PIK3CG [43]. Taselisib may be a potential treatment strategy for METTL2A high expression ER-positive BRCA patients. Furthermore, we identified another compound, Trifluridine, which interferes with DNA synthesis and inhibits cell proliferation [44, 45]. PD407824 is a CHK1 and WEE1 inhibitor which associated with METTL2A regulated gene network of DNA synthesis and cell proliferation in BRCA tumors. Taken together, METTL2A maybe a potential new therapeutic target for BRCA treatment. Nevertheless, future in-depth research and clinical studies are warranted to substantiate our findings.

## Conclusion

This study expands our knowledge of m3C modification-associated genes in pan-cancer. Importantly, we found that the expression level of METTL2A might be a biomarker for the prognosis of BRCA tumors. Three drugs including Taselisib, Trifluridine, and PD407824 showed good predictive potential in BRCA-positive patients with METTL2A overexpression.

## Supplementary Information

**Additional file 1:** **Fig. S1** Aberrant expression of METTL2A in BRCA from GEO cohorts. a, b The expression alteration of METTL2A probes between tumor (red) and normal (blue) tissue samples in BRCA from GSE3744. c The Overall survival curve of METTL2A in GSE1456. d The Disease-Free survival curve of METTL2A in GSE4922. e, f The Relapse-Free survival curves of METTL2A in e GSE1456 and f GSE6532.

**Additional file 2:** **Fig. S2** Relationship between METTL2A expression and clinicopathological parameters in BRCA form GEO cohorts. Distribution of METTL2A expression stratified by a, b Age, c N stage, d M stage, and e-g PR status.

## Data Availability

All the datasets used in our analysis are publicly available and all web links are described in the “Methods” section. All codes and processed data were available on Github (https://github.com/Hannisal/M3C.git).

## Abbreviations

METTL2A: Methyltransferase 2A
METTL2B: Methyltransferase 2B
METTL6: Methyltransferase 6
METTL8: Methyltransferase 8
TCGA: The cancer genome atlas
BRCA: Breast cancer
DDRA: Differential drug response analysis
m3C: N3-methylcytidine
GEO: Gene expression omnibus
RMA: Robust multi-array average
CNV: Copy number variation
CPTAC: Clinical proteomic tumor analysis consortium
IHC: Immunohistochemistry
GO: Gene ontology enrichment analysis
GSEA: Gene set enrichment analysis
