# Supplementary figures and images for: Integrative analysis of m3C associated genes reveals METTL2A as a potential oncogene in breast Cancer
Source: J Transl Med. 2022 Oct 20;20:476. doi: 10.1186/s12967-022-03683-2 (PMC9583565; doi:10.1186/s12967-022-03683-2)

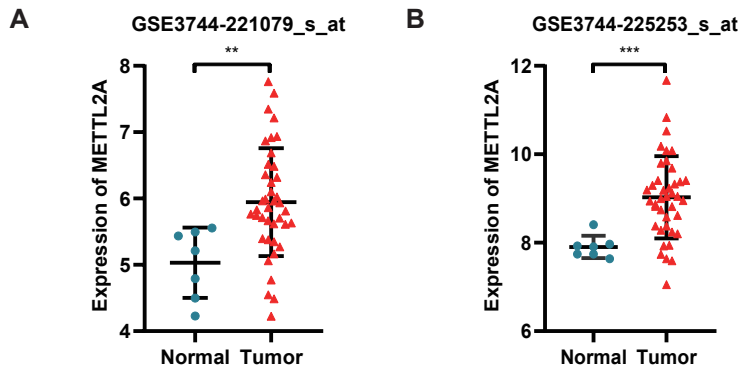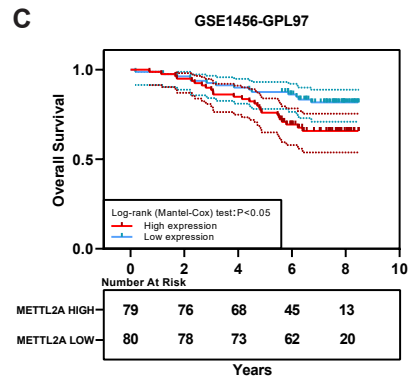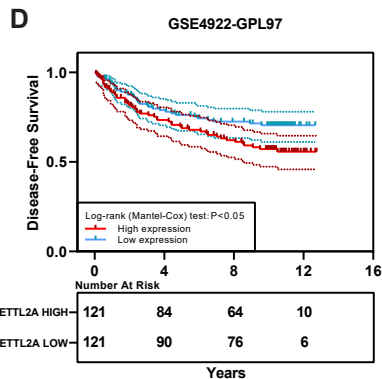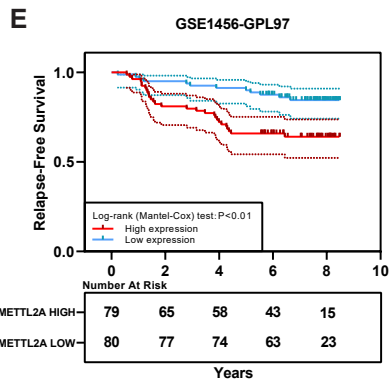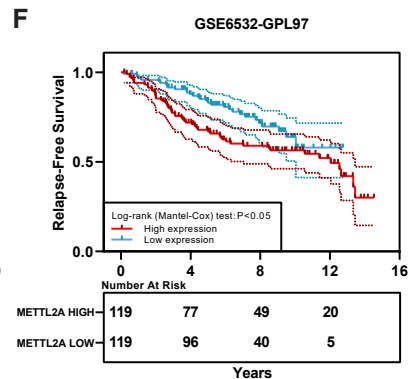

Supplement: Supplementary file 1 — Additional file 1: Fig. S1 Aberrant expression of METTL2A in BRCA from GEO cohorts. a, b The expression alteration of METTL2A probes between tumor (red) and normal (blue) tissue samples in BRCA from GSE3744. c The Overall survival curve of METTL2A in GSE1456. d The Disease-Free survival curve of METTL2A in GSE4922. e, f The Relapse-Free survival curves of METTL2A in e GSE1456 and f GSE6532. [file 12967_2022_3683_MOESM1_ESM.pdf]

**A**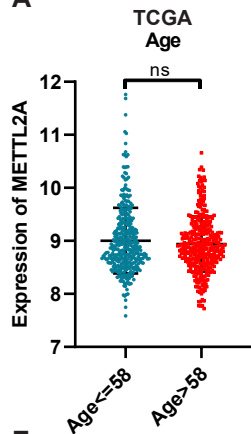**B**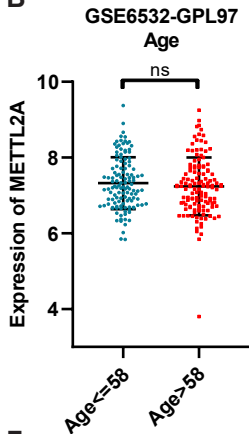**C**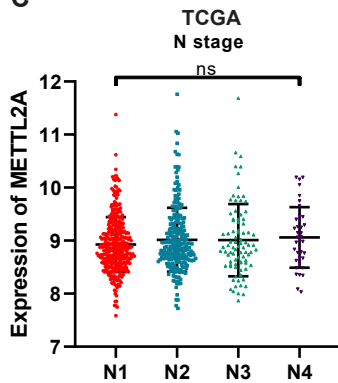**D**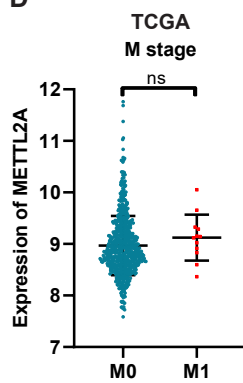**E**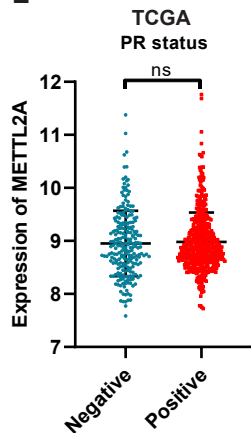**F**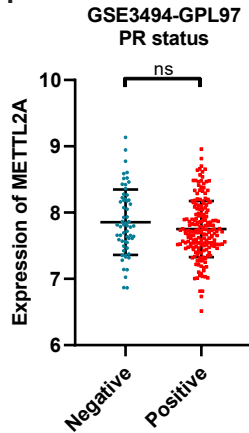**G**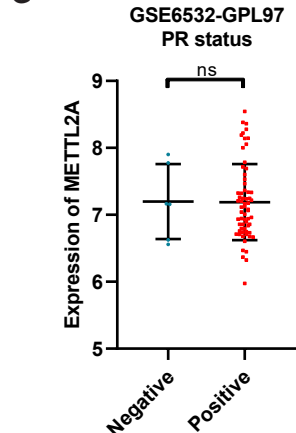

Supplement: Supplementary file 2 — Additional file 2: Fig. S2 Relationship between METTL2A expression and clinicopathological parameters in BRCA form GEO cohorts. Distribution of METTL2A expression stratified by a, b Age, c N stage, d M stage, and e-g PR status. [file 12967_2022_3683_MOESM2_ESM.pdf]
